# The American Agriculturist

**Published:** 1880-12

**Authors:** 


### BIBLIOGRAPHICAL.

*The American Agriculturist.*—

This widely known and greatly appreciated publication, devoted to the farm, garden and household, completed its 39th volume with the December number; and its editors promise more than ever before, to travel through various parts of the whole country to study the wants and methods resulting from the local difference in soils, drops, climate, implements, customs, etc. Such energy and experience cannot fail to add greatly to the value of the volume of 1881, and render it even more useful and instructive than its predecessors. A German as well as an English edition is published under the charge of a skillful and successful cultivator and excellent writer. No other agricultural or horticultural publication can compete with the American Agriculturist in cheapness of price for the same amount of material, engraving, &c, and for the useful information its pages furnish. Its premium list contains 331 useful articles and hundreds of good books, the quality of every article being vouched for. Terms: $1.50 per annum, post free. Single number 15 cents. Publishers, Orange Judd & Co., 245 Broadway, New York.

